# The kinetics of folding of the NSH2 domain from p85

**DOI:** 10.1038/s41598-019-40480-2

**Published:** 2019-03-11

**Authors:** Lorenzo Visconti, Francesca Malagrinò, Angelo Toto, Stefano Gianni

**Affiliations:** Istituto Pasteur - Fondazione Cenci Bolognetti, Dipartimento di Scienze Biochimiche “A. Rossi Fanelli” and Istituto di Biologia e Patologia Molecolari del CNR, Sapienza Università di Roma, 00185 Rome, Italy

## Abstract

SH2 domains are protein domains that mediate protein-protein interaction through the recognition and binding of specific sequences containing phosphorylated tyrosines. The p85 protein is the regulatory subunit of the heterodimeric enzyme PI3K, an important enzyme involved in several molecular pathways. In this work we characterize the folding kinetics of the NSH2 domain of p85. Our data clearly reveal peculiar folding kinetics, characterized by an apparent mismatch between the observed folding and unfolding kinetics. Taking advantage of double mixing stopped flow experiments and site directed mutagenesis we demonstrate that such behavior is due to the cis/trans isomerization of the peptide bond between D73 and P74, being in a cis conformation in the native protein. Our data are discussed in comparison with previous works on the folding of other SH2 domains.

## Introduction

SH2 domains are conserved domains of about 100 amino acids, usually found in large multidomain proteins in modular architecture with other domains like WW, SH3 and PDZ, that have the biological primary role to recognize and bind specific sequences that contain phosphorylated tyrosine residues. Accounting for just the 0.5% of the total phosphoproteome^1^, tyrosine phosphorylation is one of the most important post-translational protein modifications, regulating key physiological processes of the cell as proliferation, survival, migration^1–4^. Tyrosine phosphorilation, in facts, usually involves effectors that take part in important molecular cascades such as Ras-MAPK, JAK-STAT and MEK/ERK pathways, so that SH2 domains possess critical role in mediating interactions of proteins associated to several diseases, like cancer, methabolic sindromes and immune system disorders^3,5–7^.

The protein p85, which is the most common regulatory subunit of the heterodimeric enzyme PI3K, controls the catalytic activity of the p110 subunit of PI3K^8,9^ and mediates its interactions with various RTKs through a physical recognition between its two SH2 domains (the NSH2 and CSH2 domains, separated by a iSH2 coiled coil region) and a consensus pYxxM sequence^10,11^. PI3Ks are a family of proteins, composed of three general classes, I II and III, that catalyze the phosphorylation of phosphatidylinositols at their 3′ position. I class PI3K is the only one able to generate the second messenger PIP_3_ (phosphatidylinositol (3,4,5) triphosphate), which has a key role in several important molecular pathways in the cell^12–14^. Both CSH2 and NSH2 domains of the p85 subunit interact with p110 when the enzyme is unbound to any substrate, with an inhibitory effect on its catalytic activity. The NSH2 together with the iSH2 region have been seen to be sufficient to inhibit the activity of p110 ^15^. When the SH2 domains recognize and bind to specific sequences with phopshotyrosine the inhibition can be released, activating the enzyme^16,17^.

The three-dimensional structure of SH2 domains is highly conserved and consists of a β sheet composed by three to seven anti-parallel β strands, flanked by two α helices^18,19^. The recognition and binding with ligands occurs with the phosphorilated sequence orthogonal to the β sheet in an extended conformation^18,20^. The electrostatic nature of the interaction is mainly provided by the negative charges carried by the oxigens of the phosphotyrosine, with hydrophobic and/or electrostatic networks that stabilize the complex and involve the residues on the C-terminal side of the phosphotyrosine^18,20^.

**Figure 1:**
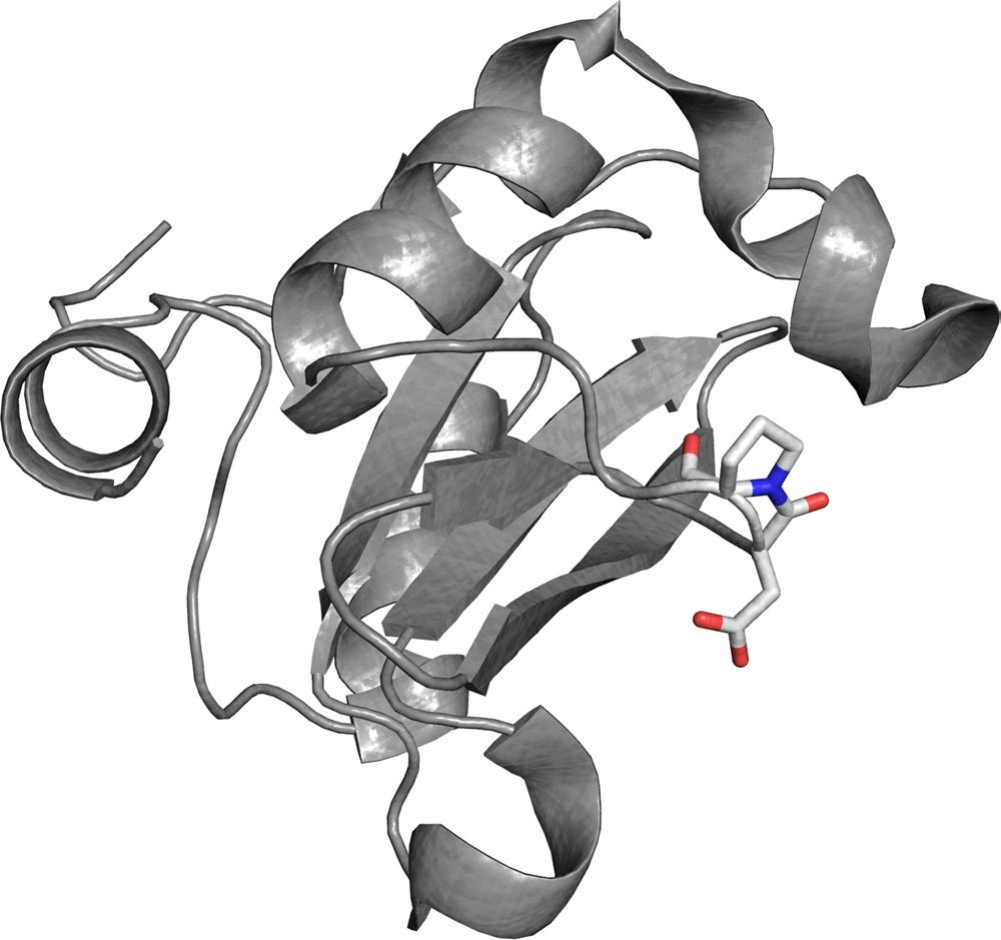
Three-dimensional structure of the NSH2 domain of p85. The residues D73 and P74, and the peptide bond in cis conformation, are highlighted in sticks.

Despite the abundant structural information available, only two SH2 domains have been characterized in their folding kinetics, intriguingly revealing different folding pathways while sharing almost superimposable three-dimensional structures^21,22^. In this paper we present a characterization of the folding of the NSH2 domain of the p85 subunit by equilibrium and kinetic experiments. The three-dimensional structure of this domain (PDB 2IUG) is homologous to the conserved fold of SH2 domains^23^, while containing a X-Pro peptide bond in a *cis* configuration in a loop between βE and βF strands (as highlighted in Fig. 1), which is not explicitly reported in the pdb file. By performing double jump stopped flow experiments, we demonstrate that the presence of the *cis*-proline has a remarkable effect on the folding of NSH2, such that the apparent complexity of the data can be easily interpreted by invoking a structural heterogeneity in the denatured state. To further support these results, we show that mutating the P74 residue into alanine restores a simple two-state mechanism of folding, confirming that the cis/trans isomerization event represents the rate limiting step in the folding of the domain.

## Results and Discussion

**Figure 2:**
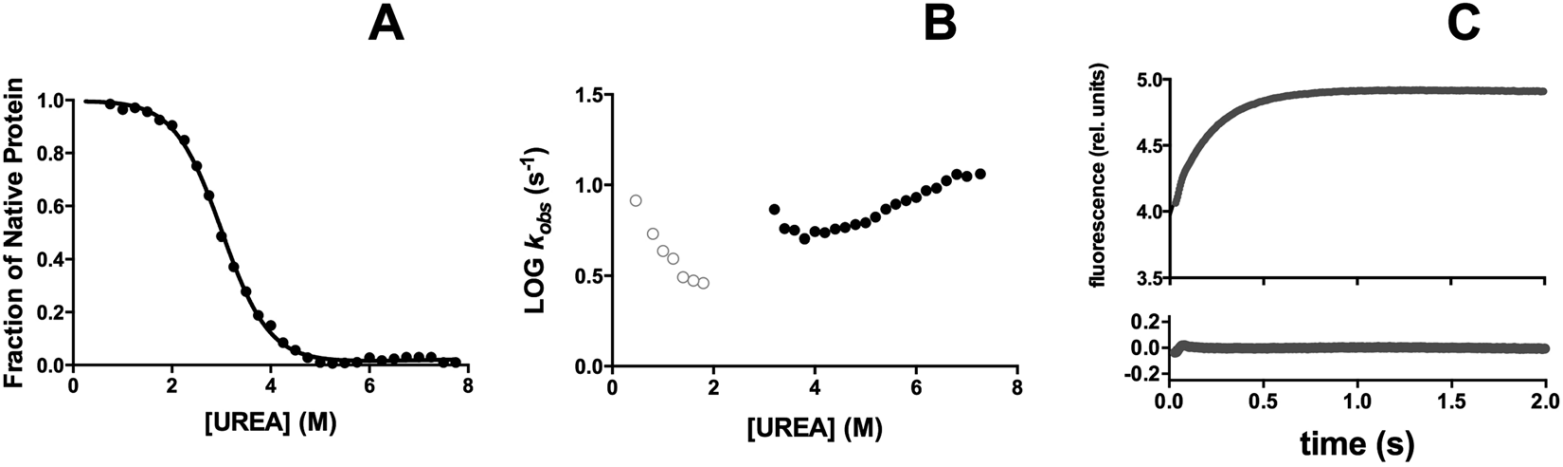
Equilibrium and kinetics of folding of wild type NSH2 domain of p85. Panel A: Equilibrium unfolding of the NSH2 domain of p85. Line is the best fit to an equation describing a two state unfolding mechanism. Panel B: Logarithm of the observed rate constants calculated from unfolding (full circles) and refolding experiments (empty circles) of NSH2 wt versus the concentration of urea. Panel C: Refolding time course of the NSH2 domain of p85 measured at 0.6 M urea at 25 °C in buffer 50 mM Hepes pH 7.0, line is the best fit to a single exponential decay.

In order to characterize the folding mechanism of the NSH2 domain of p85 we resorted to perform equilibrium and kinetic experiments. The thermodynamic stability of the protein was measured by performing equilibrium urea-induced denaturation experiments, in buffer Hepes 50 mM pH 7.0 at 25 °C by monitoring the emission change of the two tryptophan residues in positions 12 and 14, at different concentrations of urea. The decrease in fluorescence emission measured at 340 nm is reported in Fig. 2A. The observed transition suggested the presence of a simple two-state unfolding reaction, without the presence of accumulating intermediates. The unfolding free energy in water (∆G^0^) calculated was 3.6 ± 0.3 kcal mol^−1^. The m_D-N_ value, which represents the cooperativity of the unfolding reaction and is correlated with the change in the accessible surface area upon unfolding, was 1.2 ± 0.1 kcal mol^−1^ M^−1^, which is consistent with a protein of 112 residues^24^.

To study the kinetics of folding of the NSH2 domain we performed stopped-flow kinetic (un)folding experiments in buffer Hepes 50 mM pH 7.0 at 25 °C. At all the urea concentrations explored, observed kinetics were satisfactorily fitted with a single exponential equation. The logarithm of the observed rate constants obtained in unfolding (black full circles) and refolding experiments (gray empty circles) versus the concentration of urea (chevron plot) is reported Fig. 2B. A typical refolding phase is reported in Fig. 2C.

For a simple two-state folding mechanism, the unfolding and refolding arms of the chevron plot are expected to be linear, resulting in a V-shaped chevron plot^25^. However, from the inspection of Fig. 2B, it is evident that the folding and unfolding rate constants show a clear mismatch, with the *k*_*obs*_ obtained from refolding experiments being at least one order of magnitude lower than those expected from unfolding experiments. This peculiar observation demands additional investigation.

A plausible scenario to explain the unusual folding kinetics observed for NSH2 would imply a structural heterogeneity in the denatured state. In fact, if the denatured state could populate at least two distinct species with similar fluorescence and in a slow equilibrium, folding might be more complex than what expected from a simple two state scenario.

**Figure 3:**
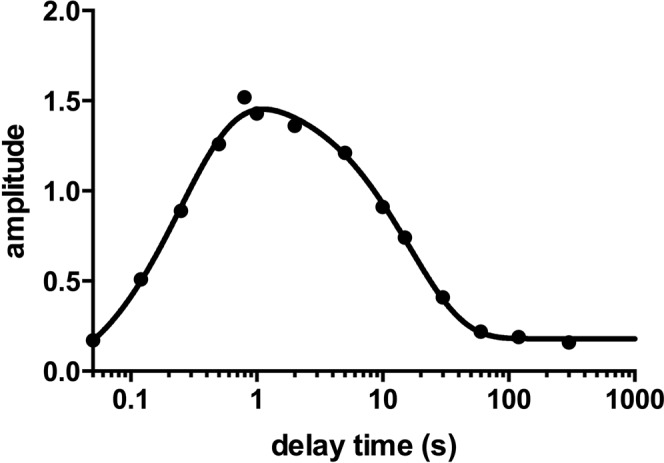
Amplitudes of the observed rate constants calculated from double mixing refolding experiments at different delay times (in logarithmic scale). Line is the best fit to a double exponential equation.

In an effort to test this hypothesis, we performed interrupted-unfolding double mixing stopped-flow experiments. In this experimental set up, the native protein was rapidly mixed with 8 M urea to initiate unfolding. Then, after different delay times (from 50 ms to 300 s), unfoding of the protein was interrupted by diluting urea in a second jump. All the refolding traces collected at different delay times were fitted with a single exponential equation. A graph of the amplitudes of the traces versus the delay time, in logarithmic scale, is reported in Fig. 3. Data were satisfactorily fitted with a double exponential equation.

**Figure 4:**
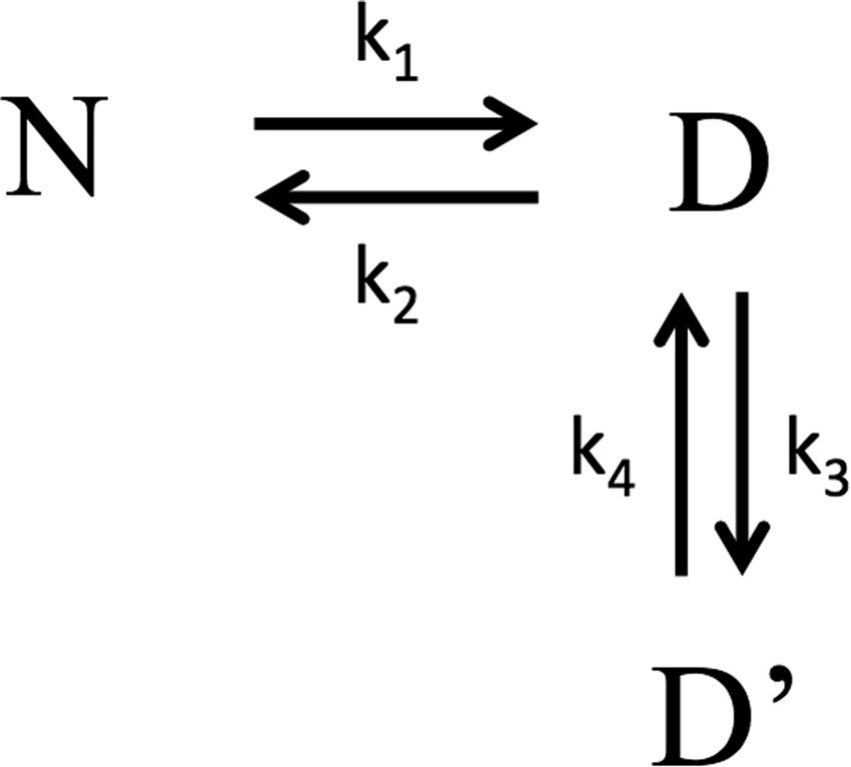
Schematic representation of the folding reaction of the NSH2 domain.

To model the data obtained by double jump experiments, we suggest the folding reaction of the NSH2 domain to be summarized as in Fig. 4, where N is the protein at its native state, and D and D’ represent two alternative conformation of the denatured state in a slow equilibrium. Short delay times (50 ms to 1 s) allowed us to monitor the formation of the state D, which unfolds before having the time to undergo a conformational change and convert to D’, and calculate the microscopic rate constant k_1_ (according to Fig. 4) which is 3.9 ± 0.3 s^−1^. Longer delay times (from 1 s to 300 s) let the protein explore the slow equilibrium between the D and D’ states. The decreasing amplitudes of the refolding traces at long delay times describe the diminishing population of proteins populating the D state in favour of the D’ state, the equilibrium being strongly shifted to the latter. The slow microscopic rate constant k_4_ (according to Fig. 4) calculated from the analysis of the amplitudes is 0.06 ± 0.01 s^−1^. NSH2 sequence presents one proline in position 74 that, in native conditions, is configured in a *cis* conformation. Data obtained from double jump experiments are compatible with a scenario in which the folding reaction of NSH2 may be slowed down by a slow equilibration between the *cis* and *trans* configuration of the P74 residue.

**Figure 5:**
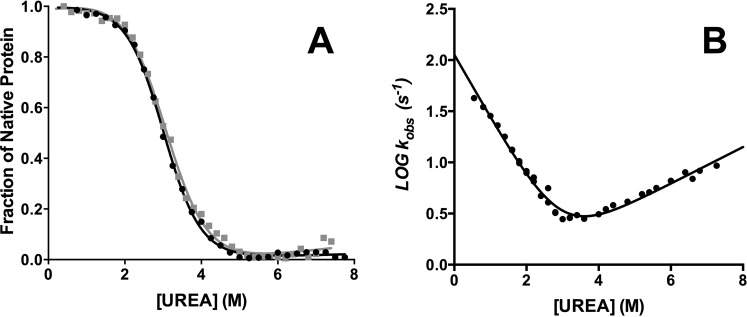
Equilibrium and kinetics of folding of the P74A mutant. Panel A: Equilibrium unfolding of wt (black circles) and P74A variant (gray squares). Lines are the best fit to an equation describing a two state unfolding mechanism. Panel B: Chevron plot of P74A variant. Line is the best fit to an equation describing a two state folding mechanism.

To validate these results, we produced the P74A variant of the NSH2 domain. Equilibrium unfolding experiment was performed. The experimental conditions were the same as for the wt. We could calculate an unfolding free energy in water (∆G^0^) of 3.7 ± 0.3 kcal mol^−1^ and a m_D-N_ value of 1.2 ± 0.1 kcal mol^−1^ M^−1^, concluding that the mutation did not affect the stability of the protein. A comparison of the wt and P74A equilibrium denaturation curves is reported in Fig. 5A. Then stopped flow kinetic folding experiments were conducted in buffer Hepes 50 mM pH 7.0 at 25 °C on the P74A variant. The resulting chevron plot is shown in Fig. 5B. Data were fitted with an equation describing a simple two-state mechanism, obtaining an excellent fit.

A powerful tool to understand the mechanism of folding of proteins is to compare the folding kinetics of proteins sharing the same structure, while displaying a different sequence. The SH2 family has been previously addressed by comparing the folding of the NSH2 domain of SHP2 with the SH2 domain of Src. While the folding of the N-SH2 domain of SHP2 revealed the presence of an intermediate accumulating in the dead time of the stopped-flow apparatus^21^, the Src SH2 domain followed a simple two-state kinetics^22^. On the basis of these previous works on SH2 domains it can be noticed that this class of proteins, despite sharing the same native structure, may explore different folding kinetics in the early stages of their folding reactions. Interestingly, apparent two-state folders can be characterized by the presence of intermediates along the folding pathway that are too unstable to be detected. This aspect is in agreement with the view that, in the early stages of the reaction, different folding pathways are selected accordingly to the primary structure, rather than the topology of the protein, determining the interactions that are crucial to define the kinetics of folding.

The folding kinetics of NSH2 of p85 appear to be highly peculiar, with an apparent mismatch between the refolding and unfolding rate constants, leading to a distortion of the chevron plot, that needed a careful additional investigation. Taking advantage of double mixing experiments as well as site-directed mutagenesis, we concluded that NSH2 folds via a simple-two state mechanism, whereby its denatured state populates predominantly an alternative conformation, characterized by a *trans* configured peptide bond between D73 and P74 residues, the *trans-to-cis* isomerization representing the rate limiting step of the folding reaction of the domain.

## Methods

### Site-Directed Mutagenesis

The construct encoding the NSH2 domain of PI3K protein was subcloned in a pET28b+ plasmid vector. The construct encoding the P74A mutant was obtained using the gene encoding the NSH2 wt as a template to perform a site-directed mutagenesis with the QuickChange Lightning Site-Directed Mutagenesis kit (Agilent technologies) according to the manufacturer’s instructions. The mutation was confirmed by DNA sequencing.

### Protein Expression and Purification

The expression of both NSH2 wt and NSH2 P74A, was performed in *E.coli* cells BL21. Bacterial cells were grown in LB medium, containing 30 μg/mL of kanamycin, at 37 °C until OD600 = 0.7−0.8, and then protein expression was induced with 1 mM IPTG. After induction, cells were grown at 37 °C overnight and then collected by centrifugation. To purify the protein, the pellet was resuspended in buffer made of 50 mM sodium phosphate, 300 mM NaCl, pH 7.2, with the addition of antiprotease tablet (Complete EDTA-free, Roche), then sonicated and centrifuged. The soluble fraction from bacterial lysate was loaded onto a nickel-charged HisTrap Chelating HP (GE Healthcare) column equilibrated with 50 mM sodium phosphate, 300 mM NaCl, pH 7.2. Protein was then eluted with a gradient from 0 to 0.5 M imidazole by using an AKTA-prime system. Fractions containing the protein were collected and the buffer was exchanged to 50 mM Hepes pH 7.0 by using a HiTrap Desalting column (GE Healthcare). The purity of the protein was analyzed through SDS-page.

### Equilibrium unfolding experiments

On NSH2 wt and P74A were carried out on a Fluoromax single photon counting spectrofluorometer (Jobin-Yvon, NJ, USA), by mixing the native protein with increasing urea concentrations. Experiments were performed at 25 °C, using a quartz cuvette with a path length of 1 cm, in 50 mM Hepes pH 7.0 and measuring the intrinsic tryptophan emission of the residues 12 and 14. The excitation wavelength was 280 nm and fluorescence spectra were recorded between 300 and 400 nm. Protein concentration was tipically 2 μM. Data were fitted using the Equation (1):

1 $${Y}_{obs}=({Y}_{N}+{Y}_{D})\frac{{e}^{{m}_{D-N}([urea]-{[urea]}_{1/2})}}{1+{e}^{{m}_{D-N}([urea]-{[urea]}_{1/2})}}$$

### Stopped-Flow experiments

*Single mixing folding experiments* were carried out on a Pi-star stopped-flow instrument (Applied Photophysics, Leatherhead, UK); the excitation wavelength was 280 nm and the fluorescence emission was measured using a 320 nm cut-off glass filter. In all experiments, performed at 25 °C in buffer 50 mM Hepes pH 7.0, refolding and unfolding were initiated by an 11-fold dilution of the denatured or the native protein with the appropriate buffer. For each denaturant concentration, usually 5 individual traces were averaged and in all cases the fluorescence time courses obtained was satisfactorily fitted by using a single exponential equation. Final protein concentrations were typically 4 µM. The chevron plot obtained for the P74A variant was fitted using the Equation (2)

2 $${k}_{obs}={k}_{f}\cdot \exp (-{m}_{f}\cdot [urea]/RT)+{k}_{u}\cdot \exp ({m}_{u}\cdot [urea]/RT)$$

*Double-jump interrupted unfolding experiments* were carried out on an SX18-MV stopped-flow instrument (Applied Photophysics) in 50 mM Acetate pH 4.0, 25 °C. The excitation wavelength was 280 nm and the fluorescence emission was measured using a 320 nm cut-off glass filter. Mixing ratios were 1:1 first mix and 1:1 second mix.

The calculated amplitudes were plotted as a function of delay times and fitted to a double exponential decay.
